# Kampo medicines, Rokumigan, Hachimijiogan, and Goshajinkigan, significantly inhibit glucagon-induced CREB activation

**DOI:** 10.1016/j.heliyon.2020.e03598

**Published:** 2020-03-20

**Authors:** Megumi Funakoshi-Tago, Seisho Yu, Akira Kushida, Kazusane Takeuchi, Hiroomi Tamura

**Affiliations:** a Division of Hygienic Chemistry, Faculty of Pharmacy, Keio University, 1-5-30 Shibakoen, Minato-ku, Tokyo 105-8512, Japan; b Cosmic Corporation Co., Ltd., Tomisaka Building, 7-3 Koishikawa 2-chome, Bunkyo-ku, Tokyo 112-0002, Japan

**Keywords:** Cell biology, Pharmaceutical science, Biochemistry, Molecular biology, Health sciences, Pharmacology, Type 2 diabetes mellitus, Kampo medicines, Glucagon, CREB, PEPCK

## Abstract

The pathophysiology of type 2 diabetes mellitus (T2DM) is characterized by not only insulin resistance, but also the abnormal regulation of glucagon secretion, suggesting that antagonizing the glucagon-induced signaling pathway has therapeutic potential in the treatment of T2DM. Although various Kampo medicines (traditional herbal medicines) are often utilized to ameliorate the symptoms of T2DM, their effects on glucagon signaling have not yet been clarified. In the present study, we examined the effects of nine types of representative Kampo formulations prescribed for T2DM on glucagon-induced CREB activation in HEK293T cells stably expressing glucagon receptor (Gcgr) and a hepatic cell line HepG2. Among these Kampo medicines, Rokumigan, Hachimijiogan, and Goshajinkigan significantly suppressed the glucagon-induced transactivation of the cAMP-responsive element (CRE)-binding protein (CREB) by inhibiting its interaction with CREB-binding protein (CBP), which led to a reduction in the expression of phosphoenolpyruvate carboxykinase (PEPCK) mRNA. Furthermore, among the crude drugs commonly contained in these three Kampo medicines, Rehmannia Root (Jio), Moutan Bark (Botampi), and Cornus Fruit (Shanzhuyu) exerted inhibitory effects on glucagon-induced CREB activation. Collectively, the present results provide a novel mechanism, the inhibition of glucagon signaling, by which Rokumigan, Hachimijiogan, and Goshajinkigan improve the symptoms of T2DM.

## 1 Introduction

Glucagon is best known as the counter-regulatory hormone to insulin, and normal glucose homeostasis depends largely on the balanced secretion of insulin and glucagon from pancreatic beta and alpha cells, respectively. Glucagon, secreted in response to low plasma glucose concentrations, plays a central role in the maintenance of fasting glycemic levels through its stimulatory effects on hepatic glucose production [1, 2, 3]. Glucagon exerts its effects through the activation of the glucagon receptor (Gcgr), a member of the class II G protein-coupled receptor superfamily [4]. Gcgr activation leads to signal transduction by G proteins, whereby Gsα activates adenylate cyclase, resulting in the accumulation of cyclic adenosine monophosphate (cAMP) and increased protein kinase A (PKA) activity [5, 6]. Gluconeogenesis in the liver is regulated at the transcriptional level by the control of phosphoenolpyruvate carboxykinase (PEPCK), which catalyzes the conversion of oxaloacetate to phosphoenolpyruvate [7, 8]. The expression of PEPCK is regulated by the transcription factor, cAMP-responsive element (CRE)-binding protein (CREB). In glucagon signaling, CREB is phosphorylated by PKA and the coactivator CBP augments the activity of phosphorylated CREB to activate the transcription of the *Pck1* gene encoding PEPCK [9, 10, 11, 12].

The pathophysiology of type 2 diabetes mellitus (T2DM) is characterized by not only insulin resistance and beta cell dysfunction, but also the abnormal regulation of glucagon secretion. Non-diabetic humans exhibit the postprandial suppression of blood glucagon, whereas patients with T2DM lack this suppression and have increased plasma glucagon levels. Furthermore, the findings of studies on subsets of patients with T2DM suggest that elevated glucagon secretion occurs antecedent to beta cell dysfunction [13, 14]. A recent study reported that the antagonism of Gcgr by a human monoclonal antibody, a competitive antagonist of Gcgr, significantly suppressed hepatic glucose production and improved glycemia [15]. Therefore, decreasing glucagon secretion and antagonizing glucagon signaling have potential as a therapeutic approach for T2DM.

Traditional Japanese herbal (Kampo) formulas are mixtures of the crude extracts of several herbs, each of which contains multiple components and are approved as ethical drugs [16]. Despite better blood glucose level control by Western medicines, Kampo medicines are also employed to treat the symptoms of T2DM, such as thirst, polyuria, and body weight loss [17]. Although nine types of Kampo medicines, Rokumigan, Hachimijiogan, Goshajinkigan, Ninjinyoueito, Juzentaihoto, Sokeikakketsuto, Byakkokaninjinto, Keishibukuryogan, and Keishikajutsubuto, are prescribed to ameliorate the symptoms of T2DM, their effects on the glucagon signaling pathway have not yet been investigated. In the present study, we examined the effects of these Kampo medicines on glucagon-induced CREB activation and found that Rokumigan, Hachimijiogan, and Goshajinkigan exerted inhibitory effects on glucagon signaling.

## 2 Materials and methods

### 2.1 Reagents

Glucagon and H-89 were purchased from the PEPTIDE INSTITUTE, INC. (Osaka, Japan) and Cyman Chemical (Ann Arbor, MI, USA), respectively. Aprotinin, pepstatin, and leupeptin were purchased from Sigma-Aldrich (St. Louis, MO, USA). Other chemicals were purchased from Nacalai Tesque (Tokyo, Japan). An anti-glucagon receptor antibody was purchased from Abcam (Cambridge, UK). An anti-phospho-CREB antibody (Ser133), anti-CREB antibody, and anti-CBP antibody were purchased from Cell Signaling Technology (Danvers, MA, USA). An anti-lamin B antibody and anti-β-actin antibody were purchased from Santa Cruz Biotechnology (Santa Cruz, CA, USA). Peroxidase-conjugated rabbit anti-mouse, rabbit anti-goat, and goat anti-rabbit secondary antibodies were purchased from Dako-Japan (Tokyo, Japan).

### 2.2 Preparation of solutions of Kampo medicines and crude drugs

Nine types of Kampo formulations, Rokumigan, Hachimijiogan, Goshajinkigan, Ninjinyoueito, Juzentaihoto, Sokeikakketsuto, Byakkokaninjinto, Keishibukuryogan, and Keishikajutsubuto, and 6 types of crude drugs, Moutan Bark (Botampi), Cornus Fruit (Shanzhuyu), Poria Sclerotium (Bukuryo), Dioscorea Rhizome (Sanyaku), and Alisma Tuber (Takusha), were purchased from Fujido Kampo Yakkyoku (Tokyo, Japan). Each Kampo formulation and crude drug were boiled in 420 ml of purified water for 30 min to prepare 300 ml of Kampo solutions using a Chinese medicine decoction device. Solutions were then desiccated by centrifugal concentration using AES2000 Automatic Environmental Speedvac (Savant, Long Island City, NY, USA) and reconstituted in the same amount of cell culture medium. We used these solutions as 100%.

### 2.3 Cell culture

HEK293T cells and HepG2 cells were purchased from the Riken Cell Bank (Ibaraki, Japan). HEK293T cells were transfected with 2 μg of the CRE luciferase vector (pNL[NlucP/CRE/Hygro]) (Promega, Madison, WI, USA) and 2 μg of the pcDNA3.1 Zeo (+) or pcDNA3.1 Zeo (+) –human glucagon receptor using Lipofectamine 2000 (Invitrogen, MD, USA). After a 24-hr incubation, cells were cultured with DMEM containing 10% fetal bovine serum (FBS) (BioWest, Nuaillé, France) and 0.25 mg/mL hygromycin B (InVivoGen, Toulouse, France), 0.25 mg/mL zeocin (InVivoGen), 100 units/ml penicillin (Nacalai Tesque), and 100 μg/ml streptomycin (Nacalai Tesque) at 37 °C and 5% CO_2_ for 2 weeks. Hygromycin B and zeocin-resistant colonies were picked up, continuously grown in selection medium, and named Mock cells and Gcgr cells, respectively. HepG2 cells were cultured with DMEM containing 10% FBS (BioWest), 100 units/ml penicillin (Nacalai Tesque), and 100 μg/ml streptomycin (Nacalai Tesque).

### 2.4 Measurement of cell viability

Mock cells and Gcgr cells (2 × 10^5^ cells) were cultured in a 24-well plate and incubated with solutions of Kampo medicines and crude drugs at 37 °C for 7 h. Cell viability was measured using the trypan blue exclusion method.

### 2.5 Luciferase assay

After Mock cells and GCGR cells (2.5 × 10^4^ cells) were cultured in a 96-well plate and incubated with solutions of Kampo medicines and crude drugs at 37 °C for 1 h and then stimulated with glucagon (10 pM) at 37 °C for 6 h HepG2 cells (4 × 10^5^ cells) were transiently transfected with 1 μg of the CRE luciferase vector using Lipofectamine 2000 (Invitrogen). After 24 h transfected HepG2 were pretreated with Kampo medicines and crude drugs at 37 °C for 1 h and stimulated with glucagon (10 pM) at 37 °C for 6 h. Luciferase Assay was performed using Nano-Glo Luciferase Assay Ststem (Promega).

### 2.6 Preparation of nuclear fractions and immunoblotting

Cells were lysed with Nonidet P-40 lysis buffer (50 mM Tris-HCl (pH 8.0), 120 mM NaCl, 1 mM EDTA (pH 8.0), 0.5% NP-40, 10 mM β-glycerophosphate, 2.5 mM NaF, 0.1 mM Na_3_VO_4_, 2 μg/mL aprotinin, and 2 μg/mL leupeptin). In order to prepare nuclear extracts, cells were lysed in buffer A (10 mM HEPES-KOH (pH 7.8), 10 mM KCl, 0.1 mM EDTA, 0.1% Nonidet P-40, 1 mM DTT, 0.5 mM PMSF, 2 μg/mL aprotinin, 2 μg/mL pepstatin, and 2 μg/mL leupeptin). Nuclei were then isolated as a precipitate by centrifugation at 5000 r.p.m. for 2 min. Isolated nuclei were lysed in Nonidet P-40 lysis buffer and homogenized using the ultrasonic homogenizer VP-50 (TAITEC, Japan). Cell lysates and nuclear extracts were then centrifuged at 15,000 r.p.m. at 4 °C for 15 min and the supernatant was mixed with Laemmli's sample buffer. Denatured samples were resolved by SDS-PAGE and transferred to polyvinylidene difluoride (PVDF) membranes (Millipore, Billerica, MA). Membranes were probed using the designated antibodies and visualized with the ECL detection system (GE Healthcare, Little Chalfont, UK) as described previously [18]. In order to show the relative phosphorylation level of CREB, the band intensities of phosphorylated CREB were normalized with the band intensities of CREB by ImageJ.

### 2.7 Immunoprecipitation

Cells were lysed in lysed with Nonidet P-40 lysis buffer and cell lysates were centrifuged at 15,000 r.p.m. for 10 min to remove debris. The supernatants were incubated with 1 μg of the anti-CBP antibody and 30 μL protein G sepharose (Zymed Laboratory, South San Francisco, CA) at 4 °C for 2 h. The immunoprecipitates were washed three times with Nonidet P-40 lysis buffer, added with Laemmli's sample buffer, and boiled at 100 °C.

### 2.8 RNA isolation and RT-PCR (reverse transcriptase-polymerase chain reaction)

RNA was prepared using Sepazol (Nacalai Tesque). RT was performed using an oligo (dT)_20_ primer (TOYOBO, Osaka, Japan) and 1 μg of total RNA for first-strand cDNA synthesis, as described previously [18]. Quantitative real-time PCR was performed using an iCycler detection system (Bio-Rad, Berkeley, CA, USA). PCR was performed in a volume of 25 μL with KAPA SYBR® FAST qPCR Kits (KAPA Biosystems, Wilmington, MA, USA). The PCR primer sequences used were as follows: PEPCK, 5′- ATCCCCAAAACAGGCCTCAG -3′ (forward) and 5′-ACGTACATGGTGCGACCTT-3′ (reverse); GAPDH, 5′- ACCACAGTCCATGCCATCAC-3′ (forward) and 5′- TCCACCACCCTGTTGCTGTA-3′ (reverse).

### 2.9 Statistical analysis

Data are expressed as averages ± SD. Statistical analyses were conducted using SPSS Statistics software (Version 23 for Macintosh, IBM Inc.). Differences were considered to be significant for values of *P* < 0.05.

## 3 Results

### 3.1 Glucagon induced the activation of CREB and expression of PEPCK mRNA in Gcgr cells

**Figure 1:**
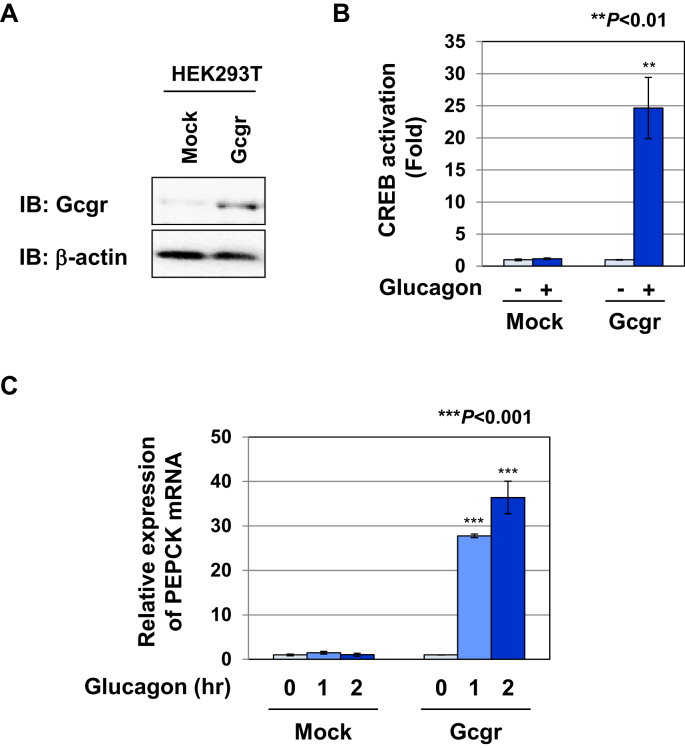
Glucagon induces the activation of CREB in HEK293T cells expressing glucagon receptor. HEK293T cells were stably transfected with the CRE luciferase reporter (CRE-Luc) and an empty vector or expression vector of glucagon receptor (Gcgr), and named Mock cells and Gcgr cells, respectively. (A) Whole cell lysates were prepared and immunoblotted with an anti-Gcgr antibody or anti-β-actin antibody. (B) Mock cells and Gcgr cells (2.5×10^4^) were stimulated with glucagon (10 pM) for 6 h. The activity of CREB was measured by a luciferase assay. Data are the mean ± S.D. of the relative luciferase activities of CRE-Luc in three experiments. ∗∗ indicates *P* < 0.01 significantly different from the group of untreated Mock cells. (C) Mock cells and Gcgr cells (1×10^6^) were stimulated with glucagon (10 pM) for the indicated periods. Total RNA was prepared and the expression of PEPCK mRNA was analyzed by quantitative real-time PCR. GAPDH mRNA was analyzed as an internal control. Values are the mean ± S.D. of three independent experiments. ∗∗∗*P* < 0.001; significantly different from the group of untreated Mock cells.

To evaluate glucagon signaling, we generated two cell strain types of HEK293T cells stably expressing the CRE luciferase reporter (CRE-Luc) and an empty vector or expression vector of the glucagon receptor (Gcgr), which were named Mock cells and Gcgr cells, respectively (Figure 1A). As shown in Figure 1B, glucagon effectively induced the activation of CREB in Gcgr cells, but not in Mock cells. Previous studies reported that the expression of PEPCK, a key enzyme for gluconeogenesis, is regulated by CREB [8, 9]. Thus, we investigated whether glucagon induced the expression of PEPCK mRNA in Mock cells and Gcgr cells by RT-PCR. Glucagon significantly induced the expression of PEPCK mRNA in Gcgr cells, but not in Mock cells (Figure 1C), suggesting that it is possible to evaluate glucagon signaling using Gcgr cells.

### 3.2 Rokumigan, Hachimijiogan, and Goshajinkigan potently inhibited glucagon-induced CREB activation in Gcgr cells

**Figure 2:**
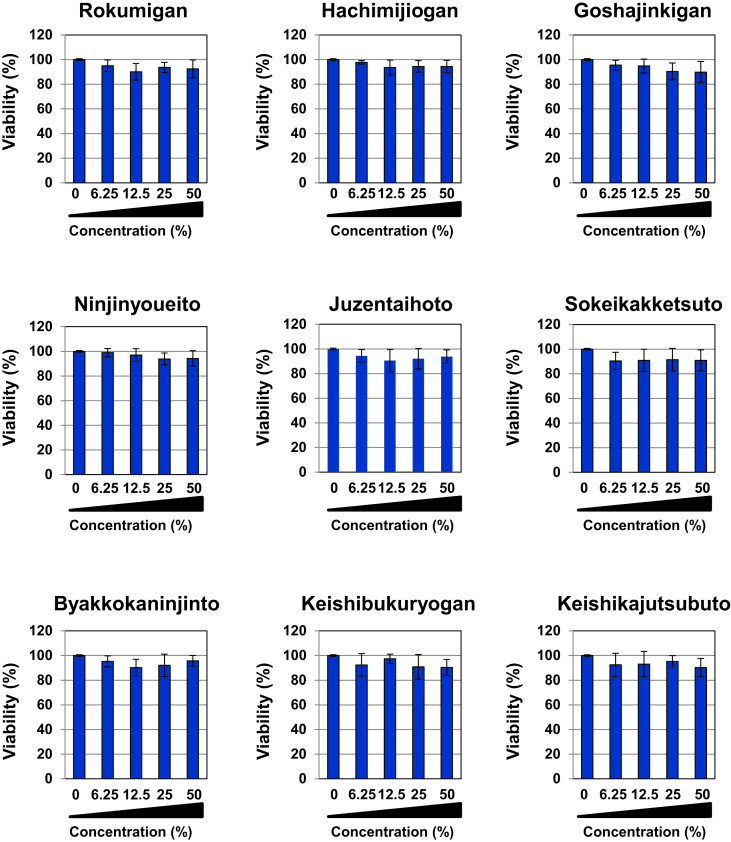
Nine types of Kampo formulations prescribed to treat type 2 diabetes have no effect on the viability in Gcgr cells. Gcgr cells (5×10^5^) were pretreated with solutions made by decocting Rokumigan, Hachimijiogan, Goshajinkigan, Ninjinyoueito, Juzentaihoto, Sokeikakketsuto, Byakkokaninjinto, Keishibukuryogan, and Keishikajutsubuto (6.25, 12.5, 25, and 50%) for 7 h. Cell viability was examined with trypan blue exclusion tests. Values are the mean ± S.D. of three independent experiments.

**Figure 3:**
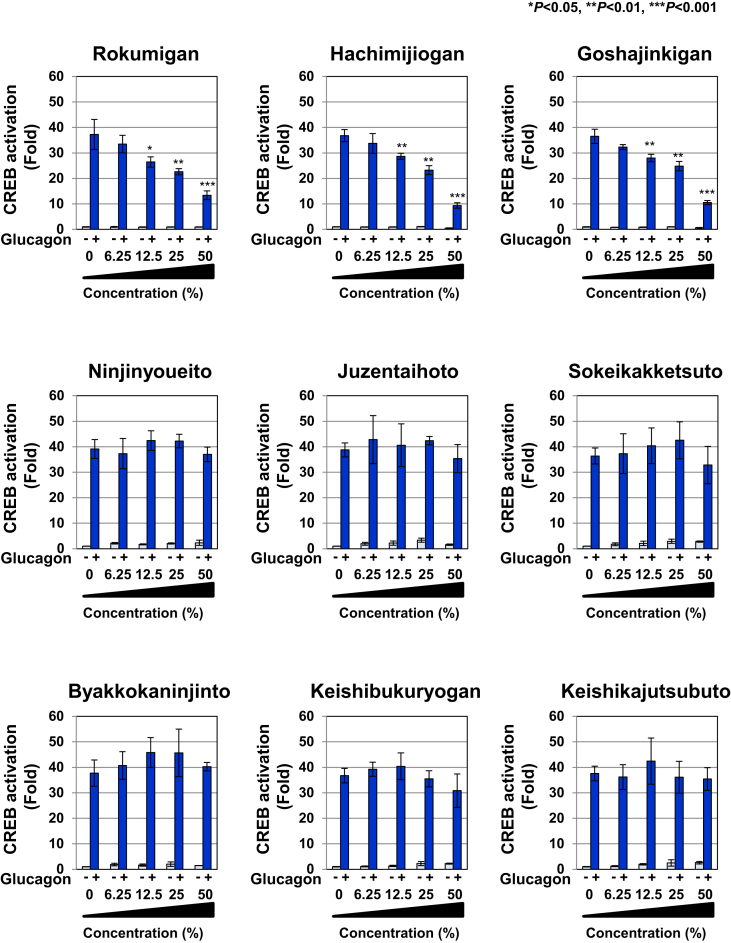
Rokumigan, Hachimijiogan, and Goshajinkigan significantly inhibit glucagon-induced CREB activation in Gcgr cells. Gcgr cells (2.5×10^4^) were pretreated with solutions made by decocting Rokumigan, Hachimijiogan, Goshajinkigan, Ninjinyoueito, Juzentaihoto, Sokeikakketsuto, Byakkokaninjinto, Keishibukuryogan, and Keishikajutsubuto (6.25, 12.5, 25, and 50%) for 1 h prior to the stimulation with glucagon (10 pM) for 6 h. The activity of CREB was measured by a luciferase assay. Data are the mean ± S.D. of the relative luciferase activities of CRE-Luc in three experiments. ∗*P* < 0.05; ∗∗*P* < 0.01; ∗∗∗*P* < 0.001 significantly different from the group stimulated with glucagon.

Nine types of Kampo medicines, Rokumigan, Hachimijiogan, Goshajinkigan, Ninjinyoueito, Juzentaihoto, Sokeikakketsuto, Byakkokaninjinto, Keishibukuryogan, and Keishikajutsubuto, are prescribed to patients with T2DM [16]. As shown in Figure 2, the viability of Gcgr cells was not affected by treatments with these Kampo drugs. The treatments with Rokumigan, Hachimijiogan, and Goshajinkigan significantly inhibited the glucagon-induced activation of CREB, whereas the treatments with Ninjinyoueito, Juzentaihoto, Sokeikakketsuto, Byakkokaninjinto, Keishibukuryogan, and Keishikajutsubuto did not (Figure 3).

### 3.3 Rokumigan, Hachimijiogan, and Goshajinkigan potently inhibited the glucagon-induced expression of PEPCK mRNA in Gcgr cells

**Figure 4:**
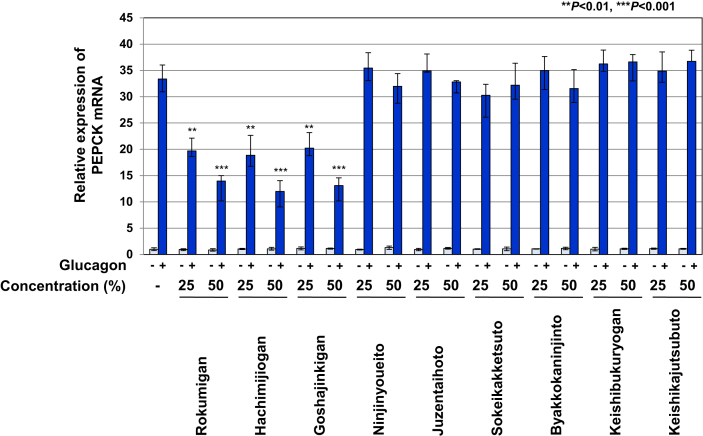
Rokumigan, Hachimijiogan, and Goshajinkigan significantly inhibit the glucagon-induced expression of PEPCK mRNA in Gcgr cells. Gcgr cells (1×10^6^) were pretreated with solutions made by decocting Rokumigan, Hachimijiogan, Goshajinkigan, Ninjinyoueito, Juzentaihoto, Sokeikakketsuto, Byakkokaninjinto, Keishibukuryogan, and Keishikajutsubuto (25 and 50%) for 1 h prior to the stimulation with glucagon (10 pM) for 2 h. Total RNA was prepared and the expression of PEPCK mRNA was analyzed by quantitative real-time PCR. GAPDH mRNA was analyzed as an internal control. Values are the mean ± S.D. of three independent experiments. ∗∗*P* < 0.01; ∗∗∗*P* < 0.001 significantly different from the group stimulated with glucagon.

We examined the effects of the nine types of Kampo drugs on the glucagon-induced expression of PEPCK mRNA in Gcgr cells. As shown in Figure 4, the treatment with Rokumigan, Hachimijiogan, and Goshajinkigan significantly inhibited the glucagon-induced expression of PEPCK mRNA in a dose-dependent manner. On the other hand, the treatment with the 6 other types of Kampo drugs had no effect on glucagon-induced PEPCK mRNA expression (Figure 4). These results suggest that the ability of Rokumigan, Hachimijiogan, and Goshajinkigan to ameliorate diabetes-related conditions is due to their inhibitory effects on glucagon-induced CREB activation.

### 3.4 Rokumigan, Hachimijiogan, and Goshajinkigan had no effect on the glucagon-induced phosphorylation of CREB in Gcgr cells

**Figure 5:**
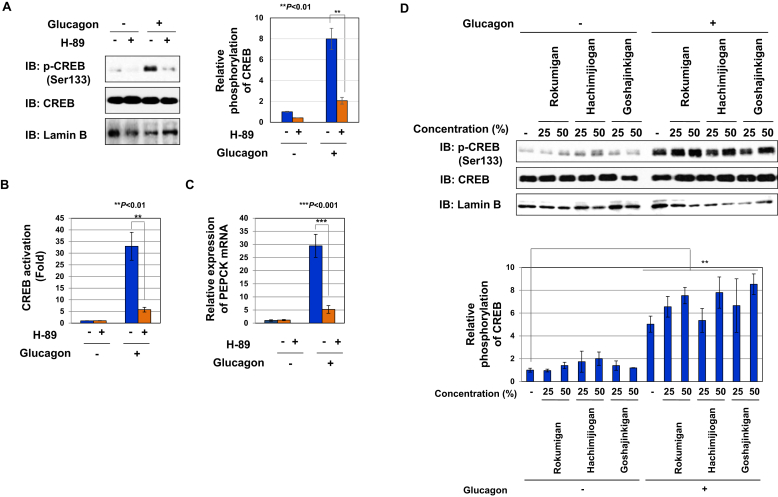
Rokumigan, Hachimijiogan, and Goshajinkigan have no effect on the PKA-induced phosphorylation of CREB in Gcgr cells. (A) Gcgr cells (1×10^6^) were pretreated with the PKA inhibitor, H-89 (25 μM) for 1 h prior to the stimulation with glucagon (10 pM) for 1 h. Nuclear extracts were prepared and immunoblotted with an anti-phospho-CREB antibody (Ser133), anti-CREB antibody, or anti-lamin B antibody. The relative phosphorylation level of CREB was graphed. Data are the mean ± S.D. of the relative phosphorylation of CREB in three experiments. ∗∗ indicates *P* < 0.01. (B) GCGR cells (2.5×10^4^) were pretreated with H-89 (25 μM) for 1 h prior to the stimulation with glucagon (10 pM) for 6 h. The activity of CREB was measured by a luciferase assay. Data are the mean ± S.D. of the relative luciferase activities of CRE-Luc in three experiments. ∗∗*P* < 0.01 significantly different from the group stimulated with glucagon. (C) Gcgr cells (1×10^6^) were pretreated with H-89 (25 μM) for 1 h prior to the stimulation with glucagon (10 pM) for 2 h. Total RNA was prepared and the expression of PEPCK mRNA was analyzed by quantitative real-time PCR. GAPDH mRNA was analyzed as an internal control. Values are the mean ± S.D. of three independent experiments. ∗∗∗*P* < 0.001 significantly different from the group stimulated with glucagon. (D) Gcgr cells (1×10^6^) were pretreated with solutions made by decocting Rokumigan, Hachimijiogan, and Goshajinkigan (25 and 50%) for 1 h prior to the stimulation with glucagon (10 pM) for 1 h. Nuclear extracts were prepared and immunoblotted with an anti-phospho-CREB antibody (Ser133), anti-CREB antibody, or anti-lamin B antibody. The relative phosphorylation level of CREB was graphed. ∗∗ indicates *P* < 0.01.

Previous studies reported that CREB activates the transcription of genes in response to phosphorylation by PKA at Serine 133 [9,10]. As shown in Figure 5A, the pretreatment with the PKA inhibitor, H-89 inhibited the glucagon-induced phosphorylation of CREB at Serine 133. In addition, the pretreatment with H-89 significantly inhibited the activation of CREB and expression of PEPCK mRNA induced by glucagon (Fig. 5B, C), confirming that the glucagon-induced phosphorylation of CREB at Serine 133 was critical for its activation. However, Rokumigan, Hachimijiogan, and Goshajinkigan failed to inhibit the phosphorylation of CREB in Gcgr cells stimulated with glucagon (Figure 5D). These results suggest that Rokumigan, Hachimijiogan, and Goshajinkigan inhibited the transcriptional activity of CREB without affecting the glucagon-induced activation of PKA.

### 3.5 Rokumigan, Hachimijiogan, and Goshajinkigan inhibited the glucagon-induced interaction of CREB with CBP in Gcgr cells

**Figure 6:**
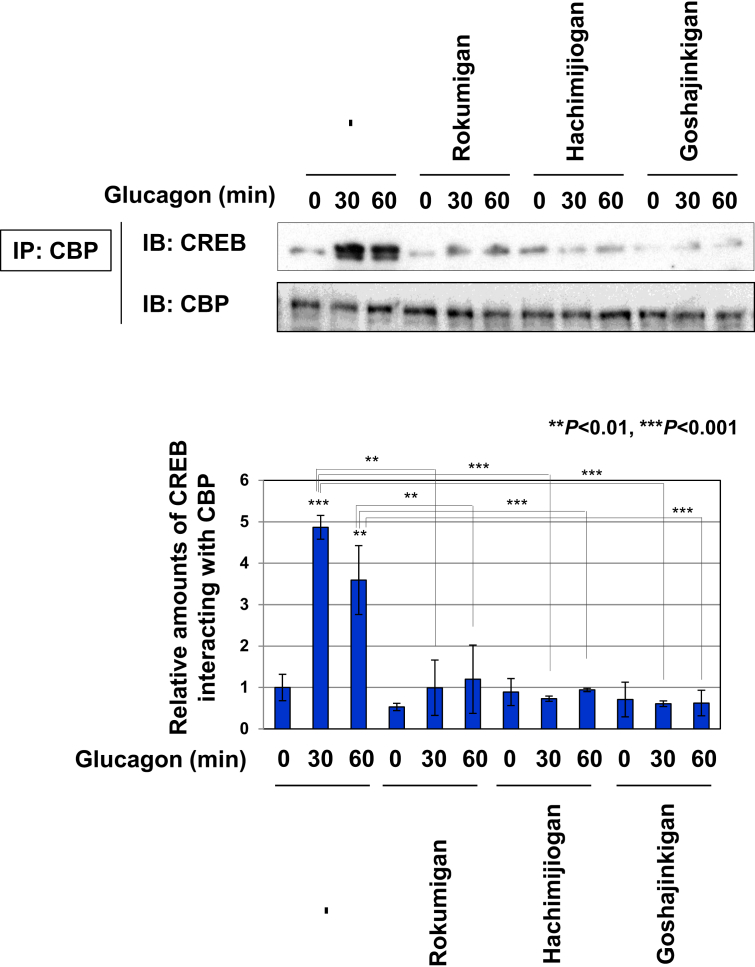
Rokumigan, Hachimijiogan, and Goshajinkigan inhibit the interaction between CREB and CBP induced by glucagon in Gcgr cells. Gcgr cells (1×10^7^) were pretreated with solutions made by decocting Rokumigan, Hachimijiogan, and Goshajinkigan (50%) for 1 h prior to the stimulation with glucagon (10 pM) for the indicated periods. Cell lysates were immunoprecipitated with an anti-CBP antibody and then immunoblotted with an anti-CREB antibody. To show the relative amounts of CREB interacting with CBP, the band intensities of CREB were normalized with the band intensities of CBP. Data are the mean ± S.D. of the relative amounts of CREB interacting CBP in three experiments. ∗∗ and ∗∗∗ indicate *P* < 0.01 and *P* < 0.001, respectively.

Previous studies demonstrated that the PKA-induced phosphorylation of CREB resulted in its binding to the co-activator CBP, leading to the augmentation of CREB transcriptional activation [11, 12]. A co-immunoprecipitation assay was performed to investigate the effects of Rokumigan, Hachimijiogan, and Goshajinkigan on the interaction between CREB and CBP in response to glucagon. CREB interacted with CBP in a glucagon-dependent manner in Gcgr cells. In contrast, the pretreatment with Rokumigan, Hachimijiogan, and Goshajinkigan markedly inhibited the glucagon-induced interaction between CREB and CBP (Figure 6). Therefore, these results suggest that Rokumigan, Hachimijiogan, and Goshajinkigan inhibited glucagon-induced CREB activation by preventing its interaction with CBP.

### 3.6 Rehmannia Root, Moutan Bark, and Cornus Fruit potently inhibited glucagon-induced CREB activation in Gcgr cells

**Figure 7:**
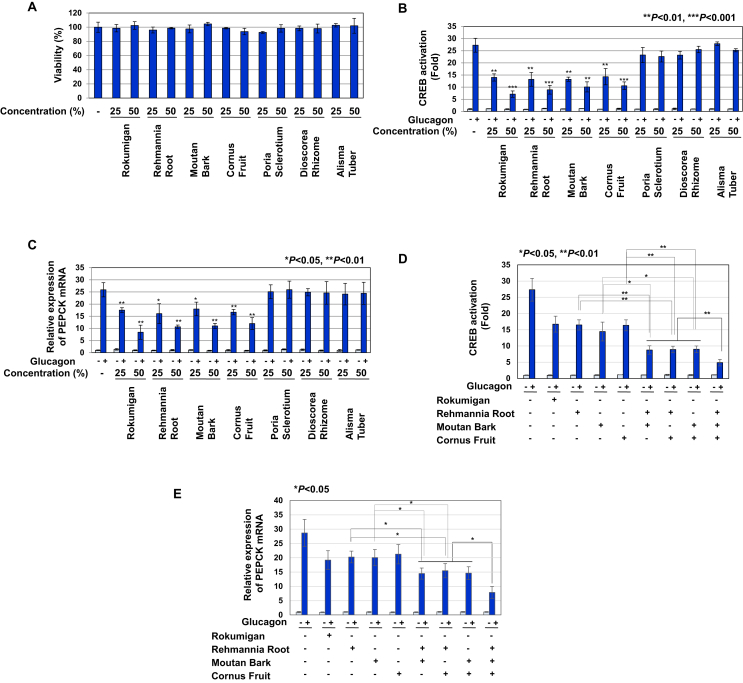
Three types of Herbal medicines, Rehmannia Root, Moutan Bark, and Cornus Fruit, significantly inhibit the glucagon-induced activation of CREB. (A) Gcgr cells (5×10^5^) were pretreated with solutions made by decocting Rokumigan, Rehmannia Root, Moutan Bark, Cornus Fruit, Poria Sclerotium, Dioscorea Rhizome, and Alisma Tuber (25 and 50%) for 7 h. Cell viability was examined with trypan blue exclusion tests. Values are the mean ± S.D. of three independent experiments. (B, C) Gcgr cells (2.5×10^4^ (B) and 1×10^6^ (C)) were pretreated with solutions made by decocting Rokumigan, Rehmannia Root, Moutan Bark, Cornus Fruit, Poria Sclerotium, Dioscorea Rhizome, and Alisma Tuber (25 and 50%) for 1 h prior to the stimulation with glucagon (10 pM) for 6 h (B) and 2 h (C), respectively. (D, E) Gcgr cells (2.5×10^4^ (D) and 1×10^6^ (E)) were pretreated with the combination of solutions made by decocting Rokumigan, Rehmannia Root, Moutan Bark, and Cornus Fruit (25 %) for 1 h prior to the stimulation with glucagon (10 pM) for 6 h (D) and 2 h (E), respectively. (B, D) The activity of CREB was measured by a luciferase assay. Data are the mean ± S.D. of the relative luciferase activities of CRE-Luc in three experiments. ∗∗*P* < 0.01; ∗∗∗*P* < 0.001 significantly different from the group stimulated with glucagon. ∗, ∗∗ and ∗∗∗ indicate *P* < 0.05, *P* < 0.01 and *P* < 0.01, respectively. (C, E) Total RNA was prepared and the expression of PEPCK mRNA was analyzed by quantitative real-time PCR. GAPDH mRNA was analyzed as an internal control. Values are the mean ± S.D. of three independent experiments. ∗ and ∗∗ indicate *P* < 0.05 and *P* < 0.01, respectively.

**Figure 8:**
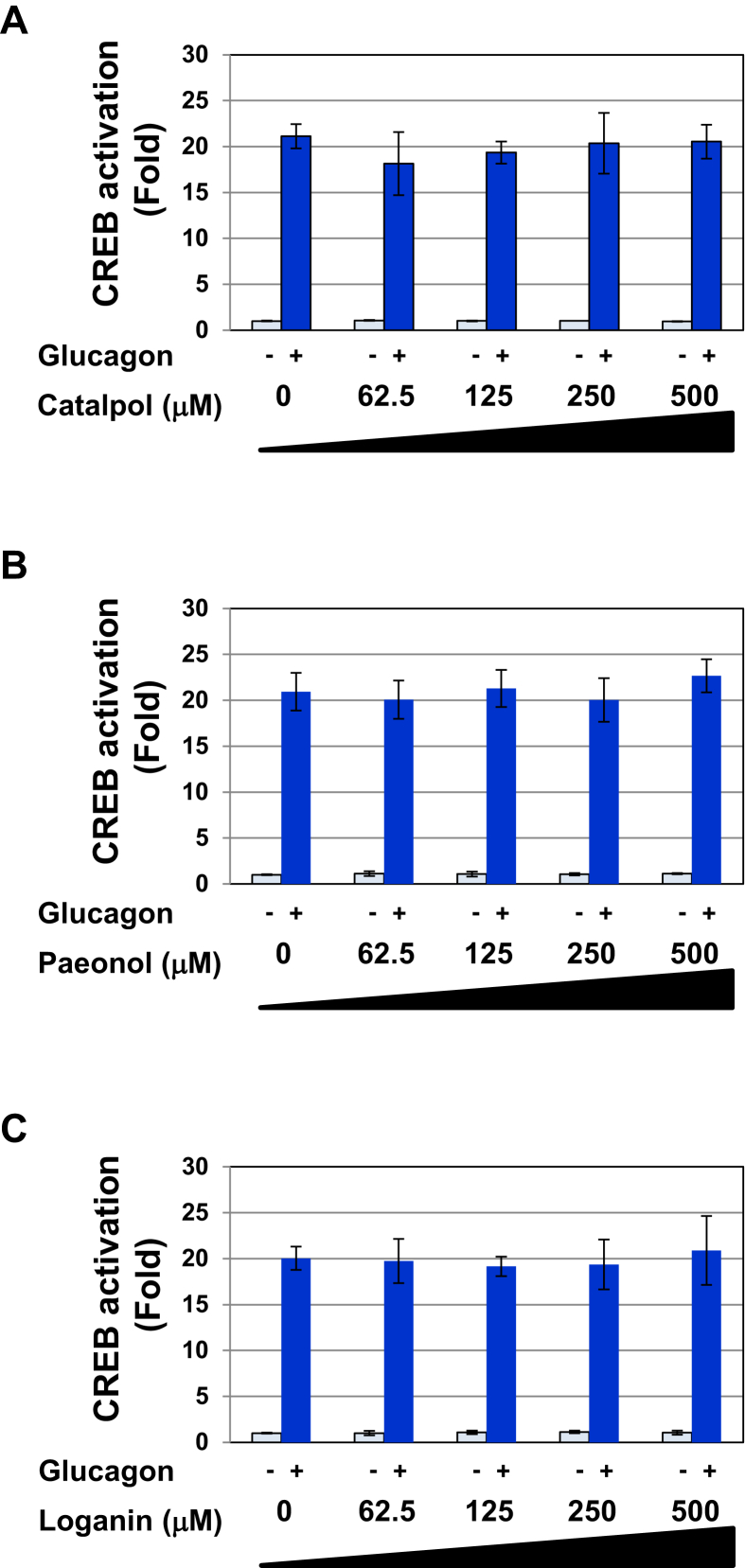
Catalpol, paeonol, and loganin have no effect on glucagon-induced CREB activation in Gcgr cells. Gcgr cells (2.5×10^4^) were pretreated with (A) catalpol, (B) paeonol, and (C) loganin (62.5, 125, 250, and 500 μM) for 1 h prior to the stimulation with glucagon (10 pM) for 6 h. The activity of CREB was measured by a luciferase assay. Data are the mean ± S.D. of the relative luciferase activities of CRE-Luc in three experiments.

The crude drugs that are common to three types of Kampo medicines, Rokumigan, Hachimijiogan, and Goshajinkigan, are Rehmannia Root (Jio), Moutan Bark (Botampi), Cornus Fruit (Shanzhuyu), Poria Sclerotium (Bukuryo), Dioscorea Rhizome (Sanyaku), and Alisma Tuber (Takusha). Every Kampo formulation contains 3 g of Moutan Bark, Cornus Fruit, Poria Sclerotium, Dioscorea Rhizome, and Alisma Tuber. Rokumigan and Hachimijiogan both contain 5 g of Rehmannia Root (Jio) and Goshajinkigan contains 6 g of Rehmannia Root (Jio). We prepared solutions of crude drugs by boiling 5 g of Rehmannia Root and 3 g each of the other crude drugs and examined the effects of the 6 types of crude drugs on glucagon-induced CREB activation. None of the crude drugs affected the viability of Gcgr cells (Figure 7A). Similar to Rokumigan, Rehmannia Root, Moutan Bark, and Cornus Fruit inhibited the glucagon-induced transactivation of CREB and expression of PEPCK mRNA in a dose-dependent manner (Fig. 7B, C). On the other hand, other crude drugs failed to inhibit glucagon-induced CREB activation and PEPCK mRNA expression (Fig. 7B, C). These results suggest that other components contained in Rehmannia Root, Moutan Bark, and Cornus Fruit are active ingredients that inhibit the glucagon signaling pathway. Although all three components and Rokumigan had similar inhibitory activity at similar dose, the treatment with combination of Rehmannia Root, Moutan Bark, and Cornus Fruit exerted additional inhibitory effect on the glucagon-induced CREB activation and PEPCK mRNA expression (Fig. 7D, E). These results suggest the possibility that the ingredients except these three components included in Rokumigan can inhibit the glucagon-induced activation of CREB. Catalpol, paeonol, and loganin are representative components contained in Rehmannia Root, Moutan Bark, and Cornus Fruit, respectively [19, 20, 21]. Therefore, we examined the effects of catalpol, paeonol, and loganin on glucagon-induced CREB activation. However, these components failed to inhibit glucagon-induced CREB activation (Figure 8).

### 3.7 Rokumigan, Hachimijiogan, Goshajinkigan, Rehmannia Root, Moutan Bark, and Cornus Fruit potently inhibited glucagon-induced CREB activation in HepG2 cells

**Figure 9:**
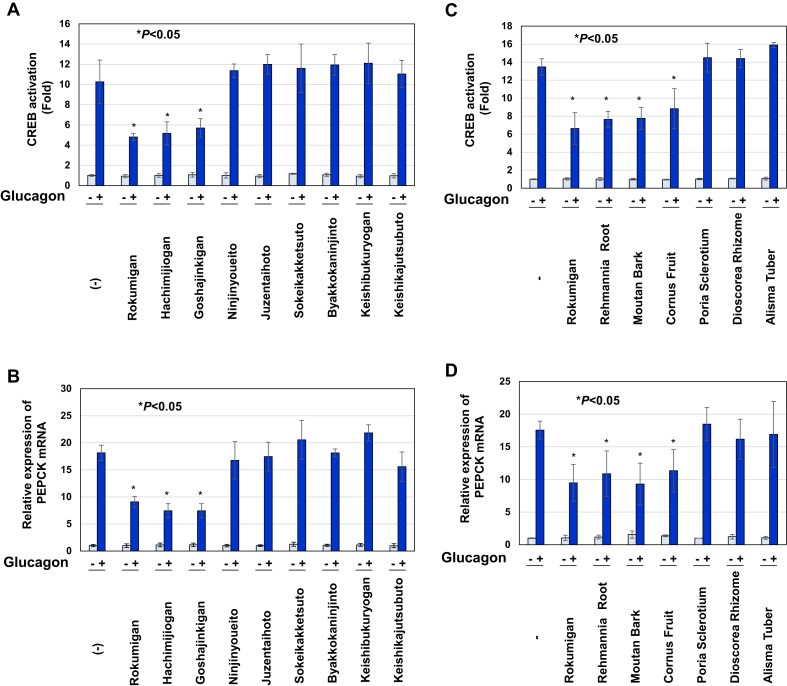
Rokumigan, Hachimijiogan, Goshajinkigan, Rehmannia Root, Moutan Bark, and Cornus Fruit significantly inhibit glucagon-induced CREB activation and PEPCK mRNA expression in HepG2 cells. (A, C) HepG2 cells (4 × 10^5^ cells) were transiently transfected with 1 μg of the CRE luciferase vector. After 24 h transfected HepG2 were pretreated with solutions made by decocting Rokumigan, Hachimijiogan, Goshajinkigan, Ninjinyoueito, Juzentaihoto, Sokeikakketsuto, Byakkokaninjinto, Keishibukuryogan, and Keishikajutsubuto (25%) (A) or with solutions made by decocting Rokumigan, Rehmannia Root, Moutan Bark, Cornus Fruit, Poria Sclerotium, Dioscorea Rhizome, and Alisma Tuber (25%) (C) for 1 h prior to the stimulation with glucagon (10 pM) for 6 h. The activity of CREB was measured by a luciferase assay. Data are the mean ± S.D. of the relative luciferase activities of CRE-Luc in three experiments. ∗*P* < 0.05 significantly different from the group stimulated with glucagon. (B, D) HepG2 cells (1×10^6^) were pretreated with solutions made by decocting Rokumigan, Hachimijiogan, Goshajinkigan, Ninjinyoueito, Juzentaihoto, Sokeikakketsuto, Byakkokaninjinto, Keishibukuryogan, and Keishikajutsubuto (25%) (B) or with solutions made by decocting Rokumigan, Rehmannia Root, Moutan Bark, Cornus Fruit, Poria Sclerotium, Dioscorea Rhizome, and Alisma Tuber (25%) (D) with for 1 h prior to the stimulation with glucagon (10 pM) for 2 h. Total RNA was prepared and the expression of PEPCK mRNA was analyzed by quantitative real-time PCR. GAPDH mRNA was analyzed as an internal control. Values are the mean ± S.D. of three independent experiments. ∗*P* < 0.05 significantly different from the group stimulated with glucagon.

**Figure 10:**
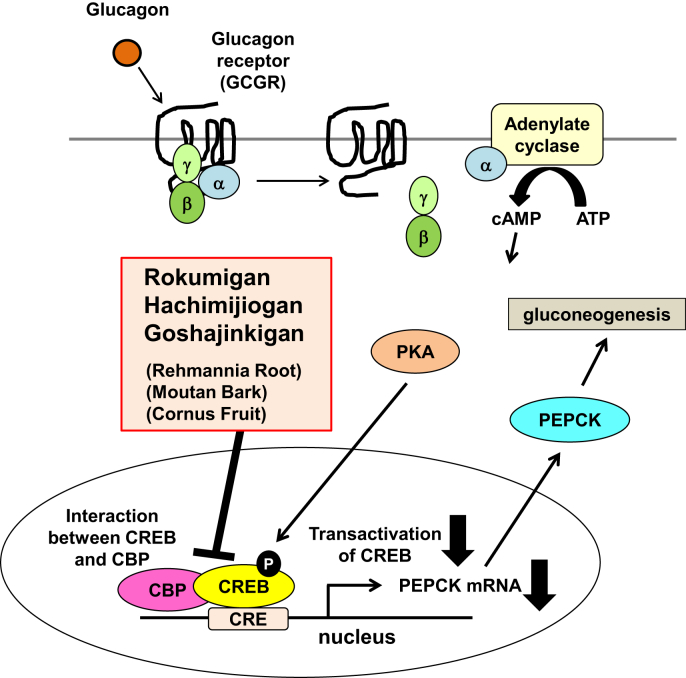
Rehmannia Root, Moutan Bark, and Cornus Fruit, which are commonly included in Rokumigan, Hachimijiogan, and Goshajinkigan, inhibit the glucagon-induced transactivation of CREB. Among the Kampo formulations prescribed to treat type 2 diabetes, Rokumigan, Hachimijiogan, and Goshajinkigan reduced the glucagon-induced expression of PEPCK mRNA by inhibiting the activation of CREB. Although these Kampo formulations had no effect on the glucagon-induced phosphorylation of CREB, they significantly inhibited the glucagon-induced transactivation of CREB by preventing the interaction between CREB and CBP. Furthermore, three types of crude drugs that are commonly included in these Kampo formulations, Rehmannia Root, Moutan Bark, and Cornus Fruit, exert inhibitory effects on the glucagon-induced activation of CREB.

We finally examined the effect of Kampo medicines and crude drugs on the glucagon-induced CREB activation and PEPCK mRNA expression using a hepatic cell line HepG2. Among nine kinds of Kampo medicines, Rokumigan, Hachimijiogan and Goshajinkigan significantly inhibited the glucagon-induced CREB activation and PEPCK mRNA expression not only in Gcgr cells but also in HepG2 cells (Figure 9A, B). We also showed that common components contained in these three Kampo medicines, Rehmannia Root (Jio), Moutan Bark (Botampi), and Cornus Fruit (Shanzhuyu), exerted inhibitory effects on glucagon-induced CREB activation and PEPCK expression in HepG2 cells (Figure 9C, D) (see Figure 10).

## 4 Discussion

In the present study, we found that Rokumigan, Hachimijiogan, and Goshajinkigan inhibited the glucagon-induced activation of CREB, leading to the inhibited expression of PEPCK mRNA, which is important in gluconeogenesis (Figure 10). Previous studies reported that Hachimijiogan and Goshajinkigan decreased blood glucose levels in diabetes model animals [22, 23, 24], and their inhibitory effects on glucagon-induced CREB activation may be attributed to reductions in blood glucose levels. Although Rokumigan, Hachimijiogan, and Goshajinkigan did not inhibit the phosphorylation of CREB in response to glucagon (Figure 5), the interaction between CREB and CBP induced by glucagon was inhibited in Gcgr cells treated with Rokumigan, Hachimijiogan, and Goshajinkigan (Figure 6). Therefore, these Kampo medicines did not appear to affect the activation of PKA, but prevented the recruitment of the transcriptional co-activator CBP to phosphorylated CREB, leading to the inhibited transactivation of CREB (Figure 9). The activity of CREB is known to be regulated by another coactivator, transducer of regulated CREB activity 2 (TORC2), in response to glucagon. Dephosphorylated TORC2 was shown to be transported to the nucleus, in which it enhanced CREB-dependent transcription. On the other hand, the AMPK-induced phosphorylation of TORC2 inhibited its nuclear accumulation [25]. Therefore, since Rokumigan, Hachimijiogan, and Goshajinkigan may inhibit the activity of AMPK, leading to the inhibition of CREB transactivation via the dephosphorylation of TORC2, it will be important to investigate the effects of these Kampo medicines on AMPK activity and the phosphorylation state of TORC2 in order to elucidate the underlying mechanisms in more detail.

By examining the influences of the crude drugs common to Rokumigan, Hachimijiogan, and Goshajinkiganon, we found that Rehmannia Root, Moutan Bark, and Cornus Fruit exerted inhibitory effects on the glucagon-induced activation of CREB, leading to the inhibition of PEPCK mRNA expression (Figures 7 and 9). The representative components, catalpol, paeonol, and loganin, contained in Rehmannia Root, Moutan Bark, and Cornus Fruit, respectively, had no effect on glucagon-induced CREB activation, suggesting that other components are active ingredients (Figure 8). The triterpene palbinone, which is included in Moutan Bark, has been shown to promote the uptake of glucose and synthesis of glycogen through the activation of AMPK in HepG2 cells [26]. A previous study also reported that ursolic acid, one of the other components of Cornus Fruit, inhibited the expression of PEPCK by 8-bromo-cAMP, an activating agent of PKA [27]. Therefore, these findings suggest that palbinone and ursolic acid inhibit the activation of CREB in response to glucagon, and further studies are needed to clarify their effects on glucagon signaling.

Previous studies showed that the administration of Byakkokaninjinto did not markedly decrease blood glucose levels in KKAy mice, a genetic animal model of diabetes mellitus [28]. Consistent with this finding, Byakkokaninjinto did not affect glucagon-induced CREB activation or PEPCK mRNA expression (Figures 3 and 4). On the other hand, the administration of Keishikajutsubuto resulted in improvements in impaired insulin effects in STZ-diabetes rats, and the administration of Keishibukuryogan produced significanUt improvements against impaired glucose tolerance in Otsuka Long-Evans Tokushima Fatty (OLETF) rats, an animal model of T2DM, suggesting that Keishikajutsubuto and Keishibukuryogan exert beneficial effects on the symptoms of T2DM [29, 30]. However, Keishikajutsubuto and Keishibukuryogan did not affect glucagon signaling (Figures 3 and 4), suggesting that these Kampo medicines exert antidiabetic effects without influencing glucagon signaling. In the present study, Rokumigan, Hachimijiogan, and Goshajinkigan significantly inhibited glucagon signaling, while the other Kampo drugs used for the purpose of the amelioration of diabetes-related conditions did not. Although we tested the effect of these Kampo medicines on glucagon action in this study, it is important to investigate the effects of these Kampo medicines on not only the glucagon action but also the glucagon production and the inhibition of insulin production on the islet cells to evaluate these Kampo medicines as suitable medicines for diabetes. However, the influences of Rokumigan, Hachimijiogan, and Goshajinkigan on the glucagon secretion have not been reported so far. Previously, it was reported that administration of Hachimijiogan significantly increased insulin secretion in type 2 diabetic model, Goto-Kakizaki (GK) rats [31]. On the other hand, Yamabe *et al.* reported that the administration of Hachimijiogan possessed a protective effect against the progression of diabetic nephropathy but had no effect on the insulin content in pancreas in a type 2 diabetic model, OLETF rats [32]. In addition, it was reported that the administration of Goshajinkigan significantly suppressed elevation in serum glucose and insulin levels in obese Zucker fatty rats (fa/fa; ZFR) [33]. The selection of appropriate Kampo medicines for the treatment of diabetes in consideration of their effects on glucagon signaling and glucagon production will be important.

## Declarations

### Author contribution statement

M. Funakoshi-Tago: conceived and designed the experiments; performed the experiments; analyzed and interpreted the data; contributed reagents, materials, analysis tools or data; wrote the paper.

S. Yu: performed the experiments; analyzed and interpreted the data; contributed reagents, materials, analysis tools or data.

A. Kushida: performed the experiments; contributed reagents, materials, analysis tools or data.

K. Takeuchi: contributed reagents, materials, analysis tools or data.

H. Tamura: conceived and designed the experiments; analyzed and interpreted the data; wrote the paper.

### Funding statement

This work was supported in part by grants from the Ministry of Education, Culture, Sports, Science and Technology (MEXT), Japan (17K08286).

### Competing interest statement

The authors declare no conflict of interest.

### Additional information

No additional information is available for this paper.
